# Monthly Summary

**Published:** 1873-07

**Authors:** 


### MONTHLY SUMMARY.

---

*A Remedy for Hæmoptysis.*—Dr. Holden desires to call the attention of the profession to a method of treatment of hæmoptysis, which, while most simple and efficacious, he has not seen described by any one ; namely, the throwing of the atomized vapor of a saturated solution of gallic acid directly into the mouth and throat. He has repeatedly found the most gratifying success to follow this treatment at once, even in cases of profuse hæmorrhage ; unlike other styptics thus administered, it quiets the spasmodic cough, which seems the direct result of the presence of the blood, requires but a moment to prepare, and aside from its efficacy, it inspires immediately the confidence of the patient. For about two years he has adopted this method, and has been surprised that no similar experience has found its way into the medical journals. His habit has been to have an atomizer and bottle gallic acid always at hand, and when summoned hastily to mix the acid in a tumbler of cold water, and use even without waiting for the excess of acid to subside. It has proved successful in several cases where blood was streaming from the mouth with every expiration.—*Med. Record.*

---

*Vehicle for Chloral Hydrate.*—J. G. Plumer, in *Lond. Pharm. Jour. and Trans.*, recommends as the best vehicle for administering chloral hydrate, the syrups flor. aurantii of the B. P. We have ourselves found nothing to answer better than essence of peppermint for disguising the peculiarly acid and nauseous taste of the drug.—*Detroit Review of Medicine.*

---

*Nervousness.*—Dr. Henry Madden, in an article on "Nervousness," takes exceptions to Dr. Bennett, who calls the sufferings of the nervous "*supposed* pains," and says "it is a distinct begging of the whole question." He proves by handy, but nevertheless true, illustrations, that the sufferings of this class of invalids are to them as severe "as are the rackings and tortures of the martyr broken on the wheel." "The difference is, that while a strong man's balance remains unmoved under a pressure of many pounds, the sensitive invalid is borne down by the weight of a feather," and in many cases that feather is necessary to do it. To further illustrate, he says: "Man's ingenuity has already enabled him to weigh the one-thousandth of a grain, to measure the one-ten-thousandth of an inch, to mark the one-ten-thousandth of a minute, to detect the one-thousandth of a degree of heat, but the unfortunate individual who feels all too keenly the hundredth part of what a strong man calls pain, is laughed at, and assured that 'it's all fancy,' and that it can easily be got rid of, if the patient will only make up his mind to forget it." To prove how the mind influences the body, he speaks of the effect of education on the special senses, and says this education is due to the "exercise of two especial functions of the mind—viz: *fixed attention* and *predominant ideas.*" To a morbidly fixed condition, or "unconscious cerebration," as Professor Laycock terms it, he thinks many of the sufferings of nervous persons are due. Indeed, he says that "the condition called nervousness may be traced to a *functional disturbance of that part of the nervous centers which presides over attention.*"—*Med. Investigator.*

---

*Oatmeal, Bone and Muscle.*—Liebig has shown that oatmeal is almost as nutritious as the very best English beef, and that it is richer than wheaten bread in the elements that go to form bone and muscle. Professor Forbes, of Edinburg, during some twenty years, measured the breadth and height, and also tested the strength of both the arms and loins of the students in the University—a very numerous class, and of various nationalities, drawn to Edinburg by the fame of his teaching. He found that, in height, breadth of chest and shoulders, and strength of arms and loins, the Belgians were at the bottom of the list; a little above them the French; very much higher, the English; the highest of all, the Scotch and Scotch-Irish, from Ulster, who, like the natives of Scotland, are fed, in their early years, with at least one meal a day of good milk and good oatmeal porridge. A very good drink is made by putting about two spoonsful of the meal into a tumbler of water. The Western hunters and trappers consider it the best of drinks, as it is at once nourishing, unstimulating and satisfying.—*Med. Investigator.*

---

*Suppression of Perspiration.*—Socoloff gives an abstract of the results which follow varnishing the skin and suppression of the cutaneous secretion:

1. A few hours before the death of the animals so treated, clonic and tetanic spasms appear in various groups of muscles, while the temperature in the rectum sinks in a marked degree.
2. Enveloping the animals in wadding did not serve to raise the temperature or arrest the fatal result.
3. Respiration of oxygen proved ineffectual to resuscitate the animals.
4. In the stomach ulcers were observed, the result of deep extravasations.
5. Albumen appeared in the urine very soon after the skin was varnished.
6. In all cases a diffuse parenchymatous inflammation of the kidneys was observed—sometimes swelling of the cells, and sometimes fatty degeneration. This result was independent of the nature of the varnish used, whether turpentine varnish, or gum.

Lang (*Arch. d. Heilkunde,* xiii., pp. 277-287, 1872) investigates the causes of death when the skin has been varnished. In addition to other phenomena he found an hour or two after death "tripple phosphate crystals" in various parts of the body, and some of the uriniferous tubules blocked with a finely granular dark mass. He thinks that the triple phosphate crystals are the result of decomposition of urea, and that the cause of death is uræmia.—*Jour. Anat. and Phys.,* November, 1872; from *Centralblatt,* No. 44, 1872.—*Am. Jour. Med.*

---

*Diarrhœa in Teething.*—In a clinical lecture "On the Primary Dentition of Children," by Francis Minot, M. D., Harvard (*Bos-*

*ton Medical and Surgical Journal*, January 2, 1873,) in speaking of the diarrhœa complicating teething during hot weather, recommends the common chalk mixture, with the addition of one-fourth part tincture of kino, which increases its astringency, and also keeps it from turning sour in hot weather. If the diarrhœa be not stopped by this mixture, one drop of laudanum may be added to a dose, but not oftener than three times a day, in children under two years old. Diarrhœa is most apt to attack children who are brought up on the bottle; hence, if the case be urgent and does not yield to treatment, a wet nurse should be procured if possible. When this cannot be done, he would strongly recommend the method of preparing the milk with arrow-root and gelatine, found in the treatise on "Diseases of Children," by Dr. Meigs and Pepper. Brandy is very useful to a teething child exhausted by diarrhœa, which should be given once in three or four hours, or oftener in urgent cases. The dose is ordinarily from five to twenty-five drops, given in milk; but if there be much prostration the physician need not fear to increase the amount.—*Canada Med. & Surg. Journal.*

---

*The Odor of Orris Root.*—This is supposed to depend on an essential oil. At a pharmaceutical meeting, in England, a British pharmaceutist, Mr. Umney, said that he had had considerable experience in regard to orris, and had no doubts in regard to the existence of an essential oil, representing the odor of the root. He had distilled many tons of the latter, and found the yield to be about one part from one thousand. The oil obtained resembled cocoa butter, and communicated an exceedingly powerful odor to alcohol. It was very expensive—more so, perhaps, than otto of roses. It was suggested by one of the members present that this oil might be the orris camphor described by Gmelin. Prof. Wayne thought the odorous principle depended more upon the soft resin than upon the oil, but this does not appear to have been the case with the oil described by Mr. Umney.—*Medical and Surgical Reporter.*

---

*Death During Etherization.*—In the *Boston Medical and Surg. Journal* of May 29, Dr. Cabot reports the following:

"An old man, weak, but not excessively so, had undergone an operation which lasted three-quarters of an hour. He was removed from the operating room, and the usual orders to watch him were given. Five hours afterwards he had a violent attack of dyspnœa, and died. Food was found in one of the bronchial tube.

"He also referred to a similar case which had occurred some time ago. A fat woman, while lying on her back under ether, vomited, and some of the vomit getting into the trachea killed her."—*Med Times.*

